# Trajectory energy minimization for cell growth tracking and genealogy analysis

**DOI:** 10.1098/rsos.170207

**Published:** 2017-05-24

**Authors:** Yin Hu, Su Wang, Nan Ma, Suzanne M. Hingley-Wilson, Andrea Rocco, Johnjoe McFadden, Hongying Lilian Tang

**Affiliations:** 1 Department of Computer Science, Faculty of Engineering and Physical Sciences, University of Surrey, Guildford, Surrey GU2 7XH, UK; 2 Department of Microbial and Cellular Sciences, School of Biosciences and Medicine, Faculty of Health and Medical Sciences, University of Surrey, Guildford, Surrey GU2 7XH, UK

**Keywords:** time-lapse image analysis, cell segmentation and tracking, *Escherichia coli*, level set framework, trajectory energy minimization

## Abstract

Cell growth experiments with a microfluidic device produce large-scale time-lapse image data, which contain important information on cell growth and patterns in their genealogy. To extract such information, we propose a scheme to segment and track bacterial cells automatically. In contrast with most published approaches, which often split segmentation and tracking into two independent procedures, we focus on designing an algorithm that describes cell properties evolving between consecutive frames by feeding segmentation and tracking results from one frame to the next one. The cell boundaries are extracted by minimizing the distance regularized level set evolution (DRLSE) model. Each individual cell was identified and tracked by identifying cell septum and membrane as well as developing a trajectory energy minimization function along time-lapse series. Experiments show that by applying this scheme, cell growth and division can be measured automatically. The results show the efficiency of the approach when testing on different datasets while comparing with other existing algorithms. The proposed approach demonstrates great potential for large-scale bacterial cell growth analysis.

## 1. Introduction

Elucidating the mechanisms underlying cell growth, division and phenotypic variation is a significant goal for biological research. Phenotypes of bacterial cells such as size, growth, division, death and genealogy can be observed by a human observer using laboratory techniques. The study of the phenotypes based on a large population can provide valuable knowledge in development and variability of cellular response to drugs [1,2]. For instance, bacterial cell growth imaging experiments can provide massive amounts of time-lapse image data of cell growth by confocal microscopy, with far more information than a human observer can digest. Therefore, it has become evident that automated segmentation and tracking are indispensable to handle and examine the large number of image data captured in even a single experiment with high efficiency, consistency and completeness.

Automated analysis of the cell growth and division process is challenging for many reasons. Firstly, the low signal–noise ratio and the large variability of image objects can make the membrane and septum of cells difficult to identify in the image. Secondly, the cell population densities are exponentially increasing due to cell elongating and dividing; this makes it hard to accurately recognize the actual shapes of cells, especially in spatially crowded clusters of cells. In addition, the challenges of cell tracking lie in the fact that the cell dynamics can cause changes of cellular morphologies, such as elongation, division, death and entering/leaving the field of view. Maintaining the consistency of segmentation and tracking results between the consecutive frames is a key objective task for many published approaches.

In previous work, to deal with the challenges discussed above, some strategies for image-based studies of cellular growth and division have been introduced, which fall into the three following basic categories. First, digital images can be manually analysed by a human with expert knowledge and skills [3]. This approach is tedious, time consuming and has a low degree of reproducibility. Second, time-lapse microscope images are fully automatically analysed by using image processing methods, such as segmentation and tracking algorithms. However, existing algorithms are not robust enough for this purpose and generate inaccurate segmentation and tracking results [4]. Third, to address the problems mentioned above, time-lapse images can be automatically processed and followed by manual editing and correcting. *Schnitzcells* [5] and *MicrobeTracker* [6] are two popular systems that can do quantitative analysis of fluorescent time-lapse images of living cells. However, such systems are laborious and not reproducible.

A comprehensive survey on the latest computational automatic analysis and software tools has been undertaken in [7]. They can be classified into two groups: tracking by detection and tracking by matching. In the first framework, cells are detected in each frame and then associations between segmented cells in consecutive sequences are established by certain criteria. This category of methods is based on the ‘first segment, then track’ scheme, as seen in [8–10]. A comparison of different cell segmentation methods has been presented in [11], where gradient features [8], cell properties [12], intensity [13,14], region accumulation and level set [15] are discussed. In addition, a review of object tracking approaches has been presented in [16], and includes sequential Monte Carlo methods [17], joint probabilistic data association filtering [18], multiple hypothesis tracking [19,20], integer programming [14], dynamic programming [21] or coupled minimum-cost flow tracking [22]. They are applied to determine the most likely cell correspondence between frames. One of the major merits for this category is its computational efficiency of segmentation stage. When only one cell is present in the field of view, the trajectory can be plausibly formed by connecting the cell location over time, and it is easier to recover from tracking failure. In addition, detection and association steps are the mutual independence, which allows straightforward tracking of new cells entering the field of view [23]. However, it is difficult to identify the real number of cells if cell densities are high, a large number of cell divisions occur, or cells enter and exit the field of view [24]. Moreover, their results are not always consistent between frames since their detection and tracking steps are mutually independent.

To avoid these problems, in the second framework, segmentation and tracking procedures are performed simultaneously. This is based on fitting a model to cells and on employing the result in the current frame as the initial points for segmentation in the next frame. This is to evolve the contours of the cells, represented either parametrically [25–27] or implicitly [28–33] using a velocity term defined by the content of the ‘target’ frame (such as gradient features, intra- and inter-region heterogeneity, shape or topology). They use morphological and behavioural clues in the model to handle the topologically flexible behaviour of cells. In addition, they try to address the changing number of cells because of cell division and dying, and cells entering or exiting the frame. The major drawback is that small errors in localization can accumulate [34]. Combining both frameworks together, Li *et al.* [30] proposed a complex cell tracking system that integrates a fast level set framework with a local association step.

Although these methods show good performance, they still have difficulties in segmenting and tracking precisely in crowded cell clusters in low-contrast images without fully identifying and recording the cell division process. To achieve these, the segmentation and tracking results should be consistent between frames. However, this is a major challenge for most of published methods. In this work, we propose an effective method to detect and track bacterial cells in large time-lapse series generated from various experiments. There are three major contributions:

— first, the profile information of cell septum and membrane is used to identify the cell division process and segment touched cells as cells exponentially grow in numbers;
— second, the global trajectory energy minimization function is developed to efficiently track cells during cell elongation and division, even if the features of cells such as length and area are changing all the time. This process can minimize the accumulated errors; and
— finally, combining local region homogeneity with the internal properties of the evolved contours (cell profile changes) can largely maintain a coherent segmentation and tracking results between the consecutive frames.

In the following sections, we describe our segmentation and tracking approach and compare it with that of *MicrobeTracker* and *MAMLE* on phase contrast images from previously published experiments and our own datasets. These published datasets were obtained growing as largely isolated cells or micro-colonies on agarose pads, which can provide high-quality images. On the contrary, our datasets show densely growing cells in the microfluidics system, which are lower-quality images but provide more information to understand their growth patterns through their life cycles. In this work, we focus on segmenting and tracking cells which are from the microfluidics system.

The rest of the paper is structured as follows. Section 2 briefly reviews the distance regularized level set evolution (DRLSE) and its minimization. Section 3 introduces the materials and the time-lapse datasets analysed in our experiments. Section 4 describes in detail our proposed scheme. Section 5 gives the experimental evaluations and discussions. The paper concludes with suggestions for future work in §6.

## 2. Edge-based active contour model in distance regularized level set evolution

In this section, we briefly review the formulation and implementation of the DRLSE proposed by Li *et al.* [35]. This method introduced a more general variational level set [36] with a distance regularization term and an external energy term that drives the motion of the zero level set towards expected locations. This model has become popular in extracting medical image contours, such as magnetic resonance imaging (MRI) images of heart [37] and computerized tomography (CT) images of brain haemorrhage [38], due to its efficiency and ability to eliminate the need for reinitialization.

Let *I* be an image on domain *Ω* and *ϕ*:*Ω*→ℜ is a level set function, an energy function E(ϕ) can be formulated as:

2.1 $$\begin{array}{rl} E(\phi ) & \;=\mu R_{p}+\lambda L_{g}+\alpha A_{g}\\ & \;=\mu \int_{\Omega }p(|\nabla \phi |)\;d\text{x}+\lambda \int_{\Omega }g\delta (\phi )(|\nabla \phi |)\;d\text{x}+\alpha \int_{\Omega }gH(-\phi )\;d\text{x}, \end{array}$$

where *p* is an energy density function p:[0,∞)∈ℜ, *δ* is the Dirac delta function [39], and *H* is the Heaviside function [40]. *μ*>0, *λ*>0 and *α*∈ℜ are the coefficients of these three terms. ∇ is the gradient operator. The edge indicator function *g* is defined by

2.2 $$g≜\frac{1}{1+|\nabla G_{\sigma }*I{|}^{2}},$$

where *G*_*σ*_ is a Gaussian kernel with a standard deviation *σ*. The convolution in equation (2.2) is used to smooth the image to reduce the surplus noise. This function *g* usually takes smaller values at expected object boundaries than at other locations.

— In equation (2.1), the first term is a penalty term $R_{p}$ to maintain the contour to evolve near the signed distance function, i.e. |∇*ϕ*|=1. Li *et al.* introduced the function *p* as:
  2.3 $$p(s)=\left\{ \begin{array}{ll} \frac{1}{{\left(2\pi \right)}^{2}}(1-\cos(2\pi s)), & \text{if}\;s\leq 1\\ \frac{1}{2}{\left(s-1\right)}^{2}, & \text{if}\;s>1. \end{array}\right.$$
— The second term $L_{g}$ in equation (2.1) calculates the line integral of the function *g* along the zero level set of *ϕ*. By parametrizing the zero level set of *ϕ* as a contour C:[0,1]→Ω, the energy can be expressed as a line integral $\int_{0}^{1}g(C(s))|C^{\prime }(s)|\;ds$ [41]. The energy is minimized when the zero level set *ϕ* is located at the desired edge of object.
— The third term $A_{g}$ in equation (2.1) calculates a weighted area of the region $\Omega_{\phi }^{-}≜\text{x}:\phi (\text{x})<0$. This energy is used to speed up the motion of the zero level set in the evolution process, which is necessary when the initial contour is set far away from the expected boundaries of the object.

The steepest descent process for minimization of the total energy functional is the following gradient flow:

2.4 $$\frac{\partial \phi }{\partial t}=\mu \;\mathrm{div}(d_{p}(|\nabla \phi |)\nabla \phi )+\lambda \delta (\phi )\;\mathrm{div}\left(g\frac{\nabla \phi }{|\nabla \phi |}\right)+\alpha g\delta (\phi ),$$

where div(⋅) is the divergence operator and *d*_*p*_ is a function defined by $d_{p}(s)≜p^{\prime }(s)/s$.

The DRLSE allows the use of more general functions as the initial contour; however, the position of the initial contour needs to be decided. If the initial contour is close to the region to be segmented, only a small number of iterations are needed to move the zero level set to the expected boundary of the object. In addition, this model can deal with the image with weak boundaries successfully.

## 3. Experiment set-ups and materials

**Figure 1.**
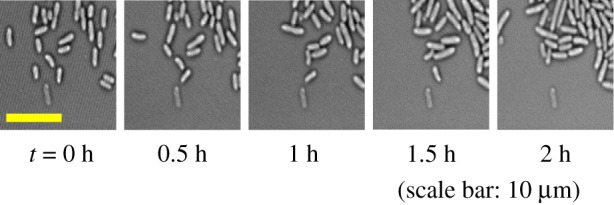
Time-lapse frame samples of our image data.

In this section, we introduce the time-lapse dataset of *Escherichia coli* (*E. coli*) cells analysed in our experiments. Figure 1 shows some sample frames from the *E. coli* dataset.

### 3.1. Bacterial strains and culturing

**Figure 2.**
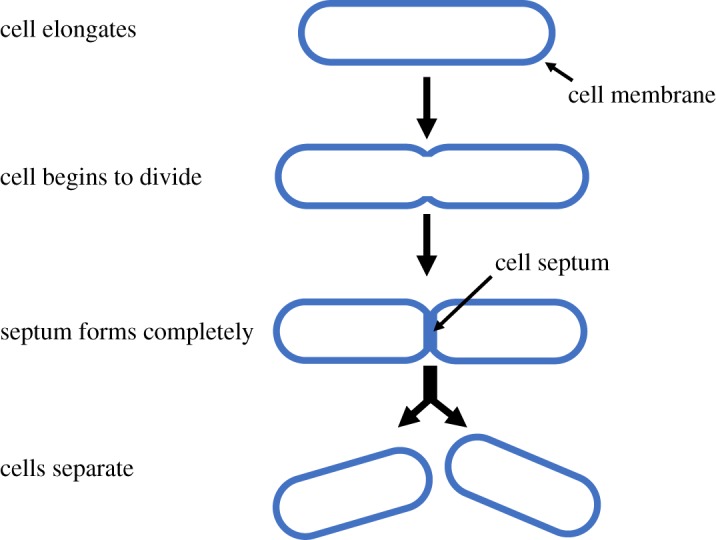
A diagram of the sequence of *E. coli* cell division.

The *E. coli* wild-type (MG1655) strain was obtained from Balaban *et al.* [42]. Figure 2 demonstrates the sequence of cell division. The formation of the septum becomes increasingly apparent during the cell growth and division. LB Lennox (Sigma Aldrich) was used for all liquid growth media, plus technical agar no. 3 (Sigma Aldrich) for plates. Ampicillin (Sigma Aldrich) was used at a final concentration of 100 μ*g* *ml*^−1^. For batch culture kill curves, aliquots were taken from an overnight culture and incubated with ampicillin for up to 48 h, at room temperature, with colony forming units measured at time 0 and then at named intervals.

### 3.2. Microfluidics platform

Table 1 gives a brief summary of acquisition set-ups and image data properties. Bacterial cells in late log phase were filtered five times through a 24 gauge needle and then loaded into a pre-warmed (37°C) microfluidic system (CellASIC ONIX Microfluidic Platform with B04A Microfluidic Bacteria Plate, with pressurized height of 0.7 μm). Media flow was controlled via the CellASIC system (which provides media exchange in less than 30 s) at a flow rate of approximately 10 μ*l* *h*^−1^. Imaging was performed under a Nikon confocal microscope (Nikon A1M on Eclipse Ti-E) equipped with an environmental chamber, motorized stage and perfect focus system.

**Table 1.** A summary of acquisition set-ups and image data properties for *E. coli* cells analysed in our experiments.

cell type	*Escherichia coli*
imaging system	Nikon A1M on Eclipse Ti-E
objective lens	40× air objective lens (Nikon Apo *λ*) with a numerical aperture of 0.95
calibration (μm px^−1^)	0.15–0.16
time step (seconds)	69
number of frames	120–140
number of series	6

Automated multi-area imaging (eight fields of view for whole growth chamber) was carried out using a 40× air objective lens (Nikon Apo *λ*) with a numerical aperture of 0.95 (which gave a sub-cellular resolution of approx. 0.32 μm). The ‘perfect focus’ system locked the cells in objective lens focal plane for the whole period of experiments with a resolution of ±25 nm. For the growth period images were taken at regular intervals of approximately 69 s (depending on experiment) to limit photo bleaching and laser damage, and imaging carried out for around 8 to 10 generations.

## 4. Proposed cell image analysis framework

Our proposed segmentation feed tracking scheme involves two steps. First, a Gaussian filtering is applied to each frame to reduce the amount of noise and enhance cell membrane and septum structures. Second, the cell regions are detected by integrating the DRLSE and object tracking frameworks simultaneously. The initial cell models generated in the previous frame are evolved in time to feed the corresponding cells in the consequent frame. With the cell elongation and division over time, clustered cells need to be split, and the mother and daughter cell relationships need to be maintained. To achieve these requirements, the DRLSE framework is integrated with a cell septum and membrane indication function that exploits a characteristic intensity profile across cells. In the rest of this section, we further explain each individual step and describe how to deal with new entering cells and unnecessary computations of the DRLSE framework.

### 4.1. Image preprocessing

**Figure 3.**
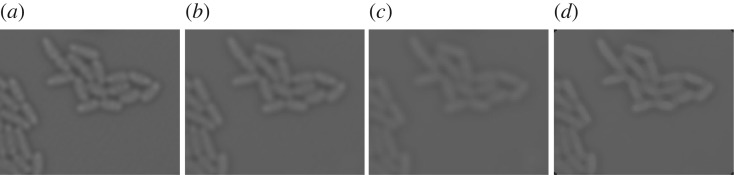
(*a*) Original image. Smoothed images after applying: (*b*) Gaussian filter with standard deviation of 1. (*c*) Gaussian filter with standard deviation of 2. (*d*) 5×5 median filter.

Since the proposed method particularly relies on the local intensity distribution to locate cell membrane and septum, it is important to reduce the effect of noise. In our implementation, we apply convolution with a Gaussian mask with a standard deviation of 1. Figure 3 shows, comparing with other kernels, that this amount of smoothing suppresses noise sufficiently while preserving true membrane and septum of cells.

### 4.2. First frame segmentation

One of the common ways is to apply a level set model to segment a single set of objects and then the level set contour is split into multiple sub-contours based on boundaries of individual objects. This approach works well in segmenting objects which have clear boundary or contrast against background. However, in our work, we need to deal with images with weak cell boundaries and with cells clustered together. To fulfil these requirements, we used one level set to evolve independently for each cell in the first frame and coevolution of a set of non-overlapping level sets for the consequent frames. The initial contours of each cell are detected and used as the ‘seeds’. These seeds move outwardly and finally stop near the boundary of the cells.

**Figure 4.**
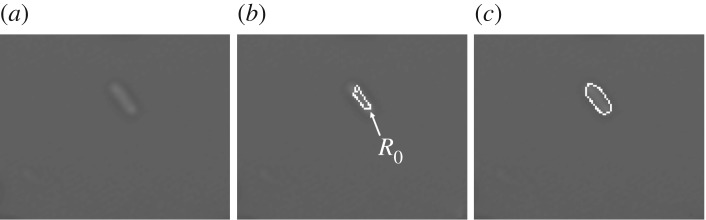
The segmentation process of the first frame. (*a*) Original input image with an isolated cell. (*b*) Preprocessed image superimposed by an initial contour *R*_0_. (*c*) Processed image superimposed by the final contour by minimizing the DRLSE framework.

Since cells are not crowded together in the first frame, their rough boundaries are easily obtained by using simple segmentation methods (such as adaptive thresholding). These boundaries are regarded as the initial contours *R*_0_ (figure 4*b*) prepared for the DRLSE framework. The final contours are detected by minimizing equation (2.1) in this framework. In places where a steady state is reached, small foreign objects (such as dust and bubbles) are eliminated from the final binary mask based on a predefined threshold *r*_*s*_. The remainder are considered as the cells to be tracked in the following frames.

### 4.3. Object tracking

One of the crucial aims of each tracking method is to preserve the identity of every tracked target over time. Since cell displacements between two successive frames are not too large, many reported approaches [28–33] used the previous cell segmentations as initial objects for the subsequent frames. However, these approaches might yield undesired results. For example, when two isolated cells in a certain frame become a cluster in the next one, it is essential to prevent their contours from merging. More importantly, to construct a cell genealogy tree, the properties of relations between mother and daughter cells need to be maintained and consistent. For instance, when one mother cell forms two isolated daughter cells in one frame, it is necessary to preserve these two isolated cell identities in the following frames. To fulfil these requirements, the DRLSE framework has been integrated with a set of indication functions.

#### 4.3.1. Object indication function

**Figure 5.**
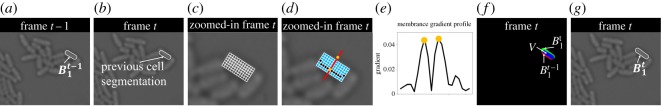
Object tracking process: (*a*) Segmented bacterial cell $B_{1}^{t-1}$ in previous frame *t*−1. (*b*) Current frame *t* is superimposed by the segmentation result $B_{1}^{t-1}$. (*c*) Split $B_{1}^{t-1}$ into small windows in current frame *t*. (*d*) In current frame *t*, classify these windows into two categories: with (small windows in black) or without information of cells’ boundaries (small windows in blue) by using intensity gradient profiles along perpendicular direction visualized in a red line. The two points in yellow indicate the edge of cell. (*e*) An intensity gradient profile. (*f*) The blue region is the intersection *V* of $B_{1}^{t-1}$ and $B_{1}^{t}$, and the pink region is the intersection of $B_{1}^{t-1}$ and the background. (*g*) The intersection *V* grows to the final boundary of *B*_1_ in frame *t*.

Maška *et al.* [31] assumed that the image can be split into a possibly disconnected background region and many possibly disconnected disjoint foreground objects. However, when a possibly disconnected disjoint foreground object contains different cells or other objects, it might yield undesired results. We have largely addressed this limitation by using cell boundary information. Assume we have obtained one cell $B_{1}^{t-1}$ in the previous frame *t*−1, and it will be used to extract the corresponding cell region $B_{1}^{t}$ in the current frame *t* by making use of the overlapped region *V* (figure 5). The intersection *V* of $B_{1}^{t-1}$ and $B_{1}^{t}$ should be first obtained and then grown to the boundary of $B_{1}^{t}$ (figure 5*g*). To obtain the overlapped region *V* , each initial object (previous segmented cell region $B_{1}^{t-1}$) is split into several small windows (*w* is the size of the window) (figure 5*c*), whereas the regions of different initial objects cannot merge together. By assigning different positive labels to individual cells, two previously separated cells can be distinguished when they touch each other. Each sub-window can merge later in time based on a homogeneity criterion:

— In the first step, these windows are classified in two different categories: with or without information on cells’ boundaries (figure 5*d*). Since these boundaries have high-intensity gradients, this information is used to identify those small regions which locate on cells’ boundaries (figure 5*e*). The windows with boundary information are eliminated to obtain multiple isolated regions. These regions might include three possible types: the intersection *V* , the intersections of $B_{1}^{t-1}$ and other cells, and the intersections of $B_{1}^{t-1}$ and background (figure 5*f*).
— In the second step, the DRLSE coevolution framework is applied to these remaining sub-windows to obtain their corresponding contours. This procedure might form some undesired contours. For instance, many contours are generated by intersection of $B_{1}^{t-1}$ and the background. Furthermore, many different cells might merge together as some sub-window regions cannot be fully isolated for a single given cell. A rod-shape and size criterion can be used to eliminate these undesired contours. We also proposed a global energy function to deal with initial cell regions expanding to different cells.
— Finally, the remaining contours are only merged within same category and same cell.

#### 4.3.2. Cell septum and membrane indication function

For each obtained cell region, a cell septum indication function is proposed to efficiently identify and record mother and daughter cells’ relationship to construct a cell trajectory and genealogy, which is often a challenging task through many published tracking approaches [7].

**Figure 6.**
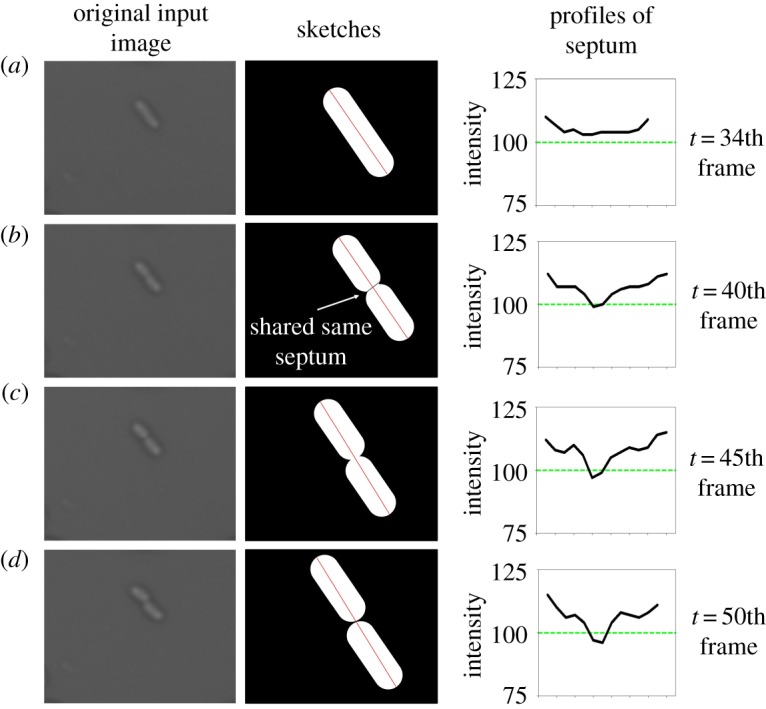
Cross-section profiles of different growing stages at different time. Red lines are the major axis of the cells, and green lines indicate the septum threshold *r*_m_. (*a*) Profile of cell when *t*=34 (single mother growing cell). (*b*) Profile of cell when *t*=40 (two daughter cells share same septum). (*c*) Profile of cell when *t*=45 (dividing into two connected daughter cells). (*d*) Profile of cell when *t*=50 (separated into two daughter cells).

**Figure 7.**
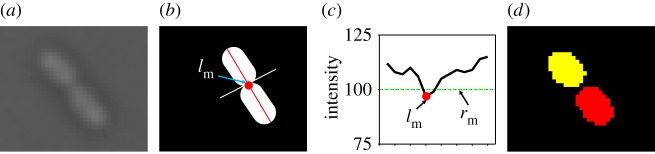
Automated separation of two connected cells. (*a*) Original input image data. (*b*) Sketch image shows local minimum (red dot), major axis (red line) and cutting line (white line). (*c*) Profile of the major axis with local minimum *l*_m_ (red dot) and threshold *r*_m_ (green line). (*d*) Segmentation result.

As a cell is growing and starting to divide, the formation of the septum becomes increasingly apparent (figure 6). The cell septum indication function exploits a characteristic intensity profile across the septum of cells to identify the stages of cell growth (red line in second column of figure 6), such as elongation and division stage. To handle cases of dividing cells which shared the same septum (figure 6*b*), we propose a criterion to automatically separate connected cells at an optimal point (figure 7). In our experiments, the local minimum of the profile was considered to be the separating point (figure 7*c*) if the value of the local minimum *l*_m_ is lower than a threshold *r*_m_. As shown in figure 7*d*, these two separated cells are regarded as two new daughter cells from their mother cell. For each frame, this threshold is denoted as *r*_m_=*μ*_septum_+*τσ*_septum_, where *μ*_septum_ and *σ*_septum_ are the mean and the standard deviation of the obtained cells’ boundary regions in the current frame, respectively. *τ* will be discussed in §5.

In addition, the width of cells is regarded as another indicator to identify the membranes in contact when a cluster of cells get crowded. If the cell width is larger than a threshold *r*_*w*_, the cells will be split by using the same strategy discussed above.

#### 4.3.3. Global trajectory energy indication function

The initial regions of one cell might expand within different cells which yields undesired results. To avoid this issue, inspired by [43], we proposed a global trajectory energy function which depends on all cells in all frames. Owing to cell elongation and division, cell properties such as length and area are not constant and cannot be used as strong cues for cell tracking. This energy function is also to address this situation. To obtain the trajectories of cells, each possible contour discussed above is used to sequentially segment and track its corresponding cells. Thus, each individual cell has different trajectories over an entire cell growing cycle from birth to division. In addition, when the cell septum indication function that defines one cell divides into two new cells, two new separate trajectories are generated.

Let the state vector **X** contain positions of all cells in all frames. ${\text{X}}_{n}^{t}$ is the location of cell *n* at frame *t*. *F* and *N* define the total number of frames and cells, respectively. The temporal length of trajectory of cell *n* is denoted by *F*(*n*):=*d*_*n*_−*b*_*n*_+1, where *b*_*n*_ and *d*_*n*_ are the first birth and final division frames, respectively. Our energy function is made up of three terms:

4.1 $$E(\text{X})=\beta E_{\mathrm{vel}}(\text{X})+\gamma E_{\mathrm{exc}}(\text{X})+\eta E_{\mathrm{reg}}(\text{X}),$$

where *β*, *γ* and *η* are weights that control the relative influence of each term.

The first term *E*_vel_(**X**) is a motion model based on a standard constant velocity assumption, which is used to reject impossible motion patterns and address ambiguities between crossing trajectories, defined by:

4.2 $$E_{\mathrm{vel}}(\text{X})=\sum_{n=1}^{N}\sum_{t=b_{n}}^{d_{n}-2}∥{\text{X}}_{n}^{t}-2{\text{X}}_{n}^{t+1}+{\text{X}}_{n}^{t+2}{∥}^{2}.$$

The second term *E*_exc_(**X**) is to penalize configurations where cells come too close to each other. In addition, this model helps to enforce unique data association since each segmented result only can be assigned to one trajectory. It is defined as:

4.3 $$E_{\mathrm{exc}}(\text{X})=\sum_{t=1}^{F}\sum_{n,j\neq n}^{N(t)}\frac{r_{w}^{2}}{∥{\text{X}}_{n}^{t}-{\text{X}}_{j}^{t}{∥}^{2}}.$$

where the scalar *r*_w_ accounts for the minimum average width of a complete cell.

The last term *E*_reg_(**X**) is to drive the optimization towards a solution for the situation in which occasionally cells keep elongating without division causing longer trajectories *N*, which is defined by:

4.4 $$E_{\mathrm{reg}}(\text{X})=N+\sum_{n=1}^{N}\frac{1}{F(n)},$$

All possible trajectory energies of each cell are calculated, and the minimum of the energies is regarded as the optimal trajectory.

### 4.4. Capturing new entering cells

It is necessary to pay attention to new cells entering into the field of view. They have not been identified in the previous frames, which possibly lead to false segmentation and tracking results at a particular frame. After segmenting and tracking the current frame without taking new entering cells into consideration, the first frame segmentation scheme is used to capture them from non-extracted cell areas. For each new entering cell, a new DRLSE function and a new trajectory is created and evolved.

### 4.5. Stopping criterion

So far, we have assumed that the minimization of the DRLSE model stops when a steady state is reached. For every iterative algorithm, it is essential to specify a suitable stopping criterion to avoid unnecessary computations when the algorithm converges. A fixed number of iterations is often the first choice, which assumes that the desired contour will be achieved after evolving the curve for a large number of iterations. However, the desired contour might have been achieved long before the iteration procedure finishes. Thus, considering the computational complexity of the level set implementations for large-scale time-lapse cell segmentations, this unnecessarily increases the execution time. Also, we must be careful so as not to terminate the iteration procedure prematurely.

**Figure 8.**
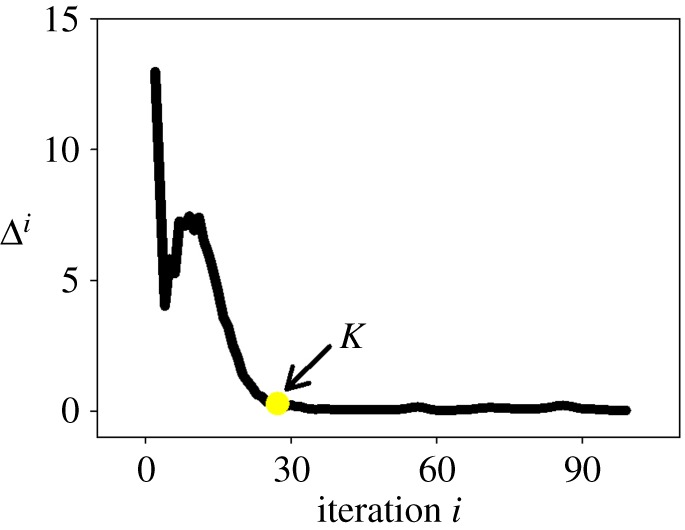
The difference between two successive iterations (Δ_*i*_) during the evolution of the DRLSE model. When Δ^*i*^≤*K* is satisfied, the minimization process of this model will stop.

In the case of our implementation, we use a general stopping criterion based on the changes of the three terms ($R_{p}$, $L_{g}$ and $A_{g}$) in equation (2.1). When the steady state is obtained, it is obvious that these three terms remain almost constant. Based on these observations, the difference between two successive iterations Δ^*i*^ is defined by:

4.5 $$\Delta^{i}=∥R_{p}^{i}-R_{p}^{i-1}{∥}^{2}+∥L_{g}^{i}-L_{g}^{i-1}{∥}^{2}+∥A_{g}^{i}-A_{g}^{i-1}{∥}^{2},\;i\geq 1,$$

where *i*−1 and *i* are two successive iterations. In the DRLSE framework, this criterion stops the curve evolution process when Δ^*i*^≤*K* is satisfied (figure 8). *K* could be used to improve the computation times as the final contour often does not change too much during the last iterations. If *K* is set to zero, the iteration stops when a steady state is reached. In our experiment, the choice of *K* will be discussed in §(b).

## 5. Experimental results and evaluation

Our proposed scheme was implemented using Matlab on a computer equipped with Intel Core i5 processor running at 2.2 GHz. Firstly, we describe the approaches to evaluate both segmentation and tracking performance, including an empirical evaluation, Dice coefficient, the multiple object tracking precision (MOTP) and the multiple object tracking accuracy (MOTA) [44]. Then, we discuss the optimal parameter settings for our scheme based on our datasets. We then compare our proposed scheme with the published tracking packages *MicrobeTracker* and *MAMLE* [45]. Meanwhile, we discuss the experimental results and highlight the differences between our proposed scheme and other developed systems.

### 5.1. Evaluation approaches

#### 5.1.1. Datasets

We evaluated the segmentation and tracking performance on three *E. coli* datasets: one from the *MicrobeTracker* example data [6], one from *Schnitzcells* [5], and one from our own dataset. All datasets were manually annotated by human experts as the ground truth. The two public datasets were obtained growing as largely isolated cells or micro-colonies on agarose pads, which can provide high-quality images comparing to our dataset. However, our dataset gave densely growing cells in the microfluidics system, which provides information when studying large-scale individual cells to understand their growth patterns through their life cycles. This dataset contains six different experiments with approximately 4000 cells. Each experiment is 180 min in length. It considers cell count as an objective metric for evaluation.

#### 5.1.2. Segmentation evaluation

According to the specifications of different approaches, cells which are undergoing division are classified as a single cell or as two independent cells. To have a fair comparison of the evaluated methods, these kinds of classifications are not considered as wrong segmentations. The results are classified into four categories:

— true positive (TP): a cell is segmented properly;
— over-segmentation: a cell is split into more than one object;
— under-segmentation: more than one cell is merged as a single cell; and
— false negative (FN): a cell is not detected.

Since false positive is a small fraction of detections (less than 0.1%) and its overall contribution is insignificant, this result is discarded from the evaluation.

To quantitatively evaluate the segmentation results, we compute the Dice coefficient between the manually annotated region (*A*) and the automatic segmented region (*B*). The Dice coefficient is computed as 2|*A*∩*B*|/(|*A*|+|*B*|).

#### 5.1.3. Multiple object tracking evaluation

Cell tracking can be regarded as a multiple object tracking algorithm. Although there are no standard evaluation approaches or datasets for multi-target tracking algorithms, the general tracking performance still can be evaluated by MOTP and MOTA. Assuming that for each time *t*, a multiple object tracker outputs a set of hypotheses {*h*_1_,…,*h*_m_} for a set of visible objects {*o*_1_,…,*o*_*n*_}, MOTP judges how well exact positions of objects are estimated by computing the Euclidean distance ${\mathrm{dis}}_{t}^{i}$ between every object *o*_*i*_ and its corresponding hypothesis.

5.1 $$\mathrm{MOTP}=\frac{\sum_{i,t}\;{\mathrm{dis}}_{t}^{i}}{\sum_{t}c_{t}},$$

where *c*_*t*_ is the number of matched object–hypothesis pairs found for time *t*.

In addition, the MOTA shows how many mistakes the tracker made in terms of misses or false negatives (FN_*t*_), false positives (FP_*t*_) and mismatches (MM_*t*_).

5.2 $$\mathrm{MOTA}=\frac{\sum_{t}({\mathrm{FN}}_{t}+{\mathrm{FP}}_{t}+{\mathrm{MM}}_{t})}{\sum_{t}g_{t}},$$

where *g*_*t*_ is the number of objects present at time *t*. In detail, the MOTA can be regarded as three error ratios: the ratio of false negatives $\bar{\mathrm{FN}}=\sum_{t}{\mathrm{FN}}_{t}/\sum_{t}g_{t}$, the ratio of false positives $\bar{\mathrm{FP}}=\sum_{t}{\mathrm{FP}}_{t}/\sum_{t}g_{t}$, and the ratio of mismatches $\bar{\mathrm{MM}}=\sum_{t}{\mathrm{MM}}_{t}/\sum_{t}g_{t}$.

### 5.2. Parameter settings

Our proposed method has 13 parameters that influence its overall performance. They can be divided into four groups based on their purposes. We used 10% of our datasets for deciding the parameters of our proposed scheme. The following gives details of various experimental set-ups as well as additional experiments to justify some of our design choices.

In the preprocessing stage, to reduce the amount of noise and preserve the rod-like structures, a Gaussian filter (*σ*=1) was performed in all experiments. The choice of *σ* relies on the width of the rod-like structures to be preserved. In addition, larger values of *σ* will smooth out the cell's membrane and septum.

The next group of parameters *r*_*s*_, *τ*, *r*_*w*_ and *w* in §(a) and (c) indicates the quantitative properties of segmented and tracked cells. They can be obtained from analysed time-lapse datasets:

— The parameter *r*_*s*_ is defined at 3 μm according to the minimum average size of a complete cell from our datasets. All objects touching the image boundary were removed.
— As a cell is growing and starting to divide, the formation of the septum becomes increasingly apparent. The parameter *τ* is defined at 1.2. When the local minimum of the septum profile is lower than the septum threshold, the connected cell is separated at the local minimum point.
— Furthermore, clustering of cells often appeared in our time-lapse datasets. Therefore, the parameter *r*_w_ was fixed at 1.2 μm to identify this situation.
— During cell tracking procedure, to ensure the sub-window includes the information of membrane, the size of sub-window should be larger than the size of membrane. The parameter *w* is defined at 0.6 μm pixels.

These parameters are converted into different values in pixel level according to the different magnification level of the microscope.

Thirdly, the most important parameters are the weights of the DRLSE framework. These are parameters *λ*, *μ* and *α* in equation (2.4), and the time step Δ_*t*_ for implementation. Li *et al.* [35] mentioned that the DRLSE model is not sensitive to the choice of *λ* and *μ*, which can be fixed for most of applications. The parameter *α* needs to be tuned for different types of images. In our work, *α* is set to a negative value to expand the contour. A non-zero *α* gives additional external force to drive the motion of contour. For a large absolute value of *α*, the contour easily passes through the object boundary in images with weak object boundaries. Therefore, we examined different parameter configurations: *λ*=1,5,…,300, *μ*=0.01,0.02,…,0.50, *α*=0,−1,…,−5 and Δ_*t*_=1,2,…,10. In the stopping criterion of the DRLSE model, the choice of *K* can speed up the computation to obtain the optimal segmentations. To obtain the optimal parameters for DRLSE model equation (2.4), the optimization scheme was performed on the evaluation dataset. Table 2 lists the top five of the parameters based on the Dice coefficient and execution time. In the following experiments, we set time step Δ_*t*_=1, *μ*=0.2, *λ*=5, *α*=−3 and *K*=0.005.

**Table 2.** Top five results of parameters in DRLSE model based on Dice coefficient and execution time.

Δ_*i*_,*μ*,*λ*,*α*,*K*	Dice coefficient	time (s)
1, 0.200, 5, −3, 0.005	0.931	125
3, 0.067, 5, −1, 0.005	0.925	112
1, 0.200, 4, −3, 0.010	0.871	105
2, 0.100, 5, −3, 0.008	0.851	182
2, 0.100, 3, −2, 0.005	0.805	98

The last group of parameters characterizes the trajectory energy function used in our proposed scheme. There are three parameters *β*, *γ* and *η* in equation (4.1). They were examined on different configurations from 0.1 to 1.0. Table 3 lists the top five of these parameters based on the MOTP and MOTA. These parameters were set to {0.02,0.5,0.5} in all our experiments.

**Table 3.** Top five results of parameters in trajectory energy function based on the multiple object tracking precision (MOTP) and the multiple object tracking accuracy (MOTA).

*β*,*γ*,*η*	MOTP (pixel)	MOTA (%)
0.02, 0.5, 0.5	92	78.36
0.02, 0.6, 0.6	91	77.56
0.03, 0.5, 0.5	141	69.58
0.01, 0.6, 0.7	155	59.62
0.04, 0.5, 0.7	222	54.94

### 5.3. Results and discussion

#### 5.3.1. Segmentation

**Figure 9.**
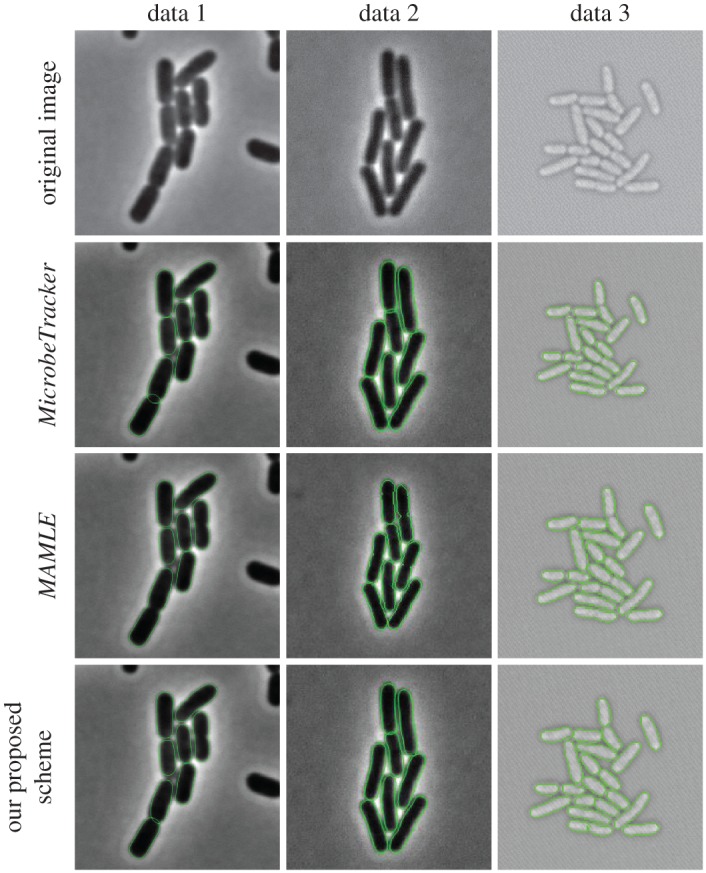
Zoomed-in segmentation results: data 1 is from the *MicrobeTracker* example data, data 2 is from *Schnitzcells* and data 3 is from our own dataset. The first row is the original images, the second row is the segmentation results of *MicrobeTracker*, the third row is the segmentation results of *MAMLE*, the last row is the segmentation results of our proposed method.

**Figure 10.**
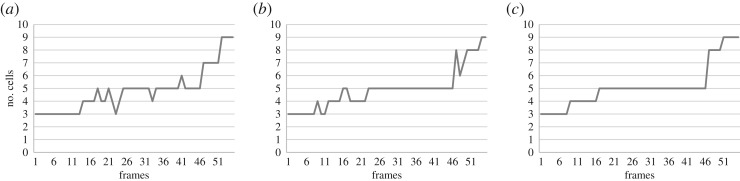
The consistency level for the number of extracted cells in sparse and medium crowded frames: (*a*) extracted cell number from the *MicrobeTracker*, (*b*) extracted cell number from *MAMLE* and (*c*) extracted cell number from our own dataset.

We compared the segmentation results of our proposed scheme with *MicrobeTracker* and *MAMLE*. It is necessary to note that neither *MicrobeTracker* nor *MAMLE* were developed for dealing with the data in microfluidics that we have. Figure 9 shows segmentation results of three different methods on *MicrobeTracker*, *Schnitzcells* and our dataset. These results demonstrated that our proposed scheme efficiently segments cells out when the septum is not clear and the membrane is too close (see third column of figure 9). To provide a compelling reason why the other two approaches are not sufficiently good for the large-scale individual cell studies, we evaluated the consistency level for the number of extracted cells in figure 10. These two previously published methods often produce non-consistent results between consecutive frames. The results in table 4 reveal the high segmentation accuracy and generality of the proposed method in different density levels, comparing to the other two methods. Illustrative examples from test samples are presented in the third column of figure 9.

**Table 4.** The segmentation accuracy and generality of the different methods in various density levels. The bold values indicate the best performance for each density level.

								segmentation
density level	no. images	no. cells (per image)	methods	TP	over-seg. (total %)	under-seg. (total %)	FN (total %)	accuracy (%)
high	158	110 600 (700)	*MicrobeTracker*	79 632	10 321 (9.33%)	9058 (8.19%)	11 589 (10.48%)	72.00%
			*MAMLE*	89 586	7201 (6.51%)	8210 (7.42%)	5603 (5.07%)	81.00%
			Our proposed scheme	95 116	6512 (5.89%)	4647 (4.20%)	4325 (3.91%)	**86.00%**
medium	272	108 801 (401)	*MicrobeTracker*	83 777	8941 (8.22%)	8201 (7.54%)	7882 (7.24%)	77.00%
			*MAMLE*	92 481	6402 (5.88%)	5004 (4.59%)	4914 (4.52%)	85.00%
			Our proposed scheme	96 833	4200 (3.86%)	4012 (3.68%)	3756 (3.45%)	**89.00%**
low	512	40 960 (80)	*MicrobeTracker*	34 816	2248 (5.49%)	1923 (4.69%)	1973 (4.82%)	85.00%
			*MAMLE*	37 683	1351 (3.29%)	879 (2.15%)	1047 (2.56%)	92.00%
			Our proposed scheme	38 912	701 (1.71%)	632 (1.54%)	715 (1.75%)	**95.00%**
total	942	260 361 (276)	*MicrobeTracker*	198 225	21 510 (8.26%)	19 182 (7.37%)	21 444 (8.24%)	76.13%
			*MAMLE*	219 750	14 954 (5.74%)	14 093 (5.41%)	11 564 (4.44%)	84.40%
			Our proposed scheme	230 861	11 413 (4.38%)	9291 (3.57%)	8796 (3.38%)	**88.67%**

Table 5 shows the comparison of the segmentation algorithms based on the proportion of cells correctly identified at different datasets at optimal Dice coefficient. It shows that the performance of our proposed scheme is comparable to that of the state-of-the-art algorithms on cells grown on agarose pads. Furthermore, it has obvious improvements on segmentation results on cells grown on microfluidics system.

**Table 5.** The proportion of correctly identified cells at optimal Dice coefficient for different methods on three datasets. The bold values are the best performance for each dataset.

	data 1	data 2	data 3
*MicrobeTracker*	78.66	70.29	63.34
*MAMLE*	**83.51**	**81.55**	78.64
Our proposed scheme	80.95	79.62	**87.56**

#### 5.3.2. Tracking

To further evaluate the performance of our proposed scheme, we tested its tracking results on a large-scale experiment. As *Schnitzcells*, *MAMLE* and *MicrobeTracker* did not provide sample video, we used our datasets to test. According to the cell density in our dataset, we chose the 100th frame (around fifth generation) as a threshold to categorize time-lapse images into medium and highly crowded density. *MAMLE* did not provide a tracking algorithm, we combined its segmentation algorithm with the tracking algorithm of *Schnitzcells*, since *MAMLE* outperforms *Schnitzcells* based on segmentation under many circumstances [46].

**Figure 11.**
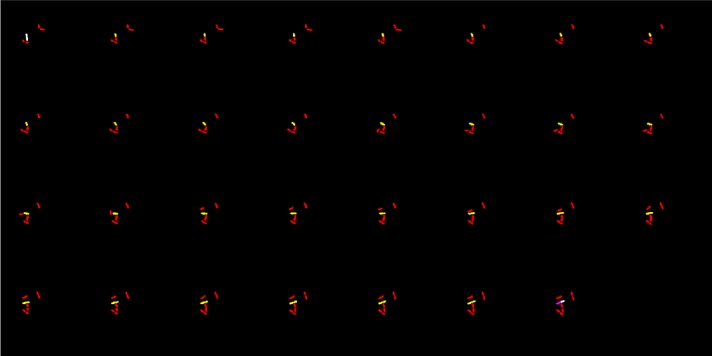
Tracking results of a single cell from its birth to division by using the proposed method. The white cell in the earliest frame is the mother of this single cell, while the yellow cells show the changes of this single cell during its growth. The last frame highlights its daughter cells of the division event shown in purple and white. Red cells are not a part of this single cell.

**Figure 12.**
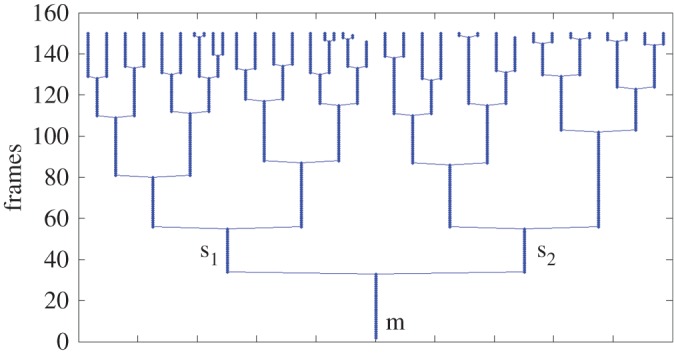
The genealogy tree of a single cell. There are around 6 generations from this single cell (m). *Y*-axis shows the number of frames in this time-lapse video. For instance, the cell (m) has existed in the first frame (frame=0). After 38 frames, cell m divides into two daughter cells (s_1_ and s_2_), and the division time for s_1_ and s_2_ are 20 frames and 18 frames, respectively.

Tables 6 and 7 illustrate the tracking performance based on precision and accuracy for datasets with medium and highly crowded density, respectively. Our proposed scheme has higher MOTA than the other two published methods in both medium and highly crowded density. In detail, our proposed trajectory energy minimization function significantly reduces the error ratio of FN, FP and MM, which are critical tasks in cell tracking systems. Figure 11 shows an illustration of the tracking results of the proposed method for a single cell. Figure 12 shows the genealogy tree of a single cell.

**Table 6.** The performance for our dataset of the group with medium crowded density based on the multiple object tracking precision (MOTP), the ratio of false negatives ($\bar{\mathrm{FN}}$), the ratio of false positives ($\bar{\mathrm{FP}}$), the ratio of mismatches ($\bar{\mathrm{MM}}$) and the multiple object tracking accuracy (MOTA). The bold values indicate the best performance on MOTP and MOTA.

	MOTP (pixel)	$\bar{\mathrm{FN}}$ (%)	$\bar{\mathrm{FP}}$ (%)	$\bar{\mathrm{MM}}$ (%)	MOTA (%)
*Schnitzcells*	**261**	10.91	1.08	18.58	69.43
*MicrobeTracker*	308	6.56	1.58	10.50	81.36
Our proposed scheme	268	5.83	0.79	8.07	**85**.**31**

**Table 7.** The performance for our dataset of the group with highly crowded density. The bold values indicate the best performance on MOTP and MOTA.

	MOTP (pixel)	$\bar{\mathrm{FN}}$ (%)	$\bar{\mathrm{FP}}$ (%)	$\bar{\mathrm{MM}}$ (%)	MOTA (%)
*Schnitzcells*	**159**	22.33	5.83	42.54	29.30
*MicrobeTracker*	181	19.81	2.49	22.50	55.20
Our proposed scheme	168	14.54	2.41	18.95	**64**.**10**

#### 5.3.3. Cell growth analysis

**Figure 13.**
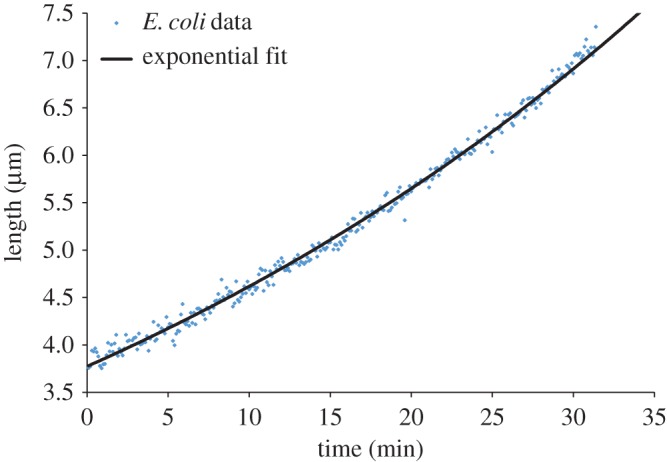
The representative growth curve of a single cell (dark blue circles).

In this work, we aimed to observe the cell growth and patterns in their genealogy. After segmenting and tracking, we analysed the size at birth, size at division, division time and elongation rate. Figure 13 reveals cells elongate approximately exponentially at the single-cell level.

## 6. Conclusion and future work

In this paper, we proposed an effective scheme to automatically segment and track *E. coli* cells in large time-lapse image series. The method incorporates the DRLSE framework with cell septum and membrane indication function and trajectory energy minimization function, which deal with cell segmentation and tracking together to perform cell genealogy analysis as well as various measures related to cell growth. The testing of our method was conducted on two published datasets and one from our own experiments. It shows that our proposed method produces competitive results, both in segmentation and tracking. Especially, it could be preferred in large-scale studies focusing on growth characteristics and genealogy of cells in which as-accurate-as-possible cell segmentation is the most desirable feature. We further analysed the cell growth over time from the birth to its division, and measured its size at birth, size at division, division time and elongation rate.

Currently, we are developing deep learning techniques to facilitate the tracking and measurement of a variety of cells. We are currently applying our proposed segmentation and tracking scheme to other bacterial cells, such as *Mycobacterium smegmatis*. Although our method works well on different density data, further improvement of this scheme will handle over-crowded data where cell shapes are irregular and not constant between consecutive frames, as the limited space available in microfluidic devices is quickly filled up by exponentially growing bacterial populations. Furthermore, we would like to speed up our proposed scheme and integrate sub-pixel precision in segmentation.

## Data accessibility

Our data are deposited at Dryad: http://dx.doi.org/10.5061/dryad.0q35m [47].
